# The Influence of Halide Ion Substitution on Energy Structure and Luminescence Efficiency in CeBr_2_I and CeBrI_2_ Crystals

**DOI:** 10.3390/ma16145085

**Published:** 2023-07-19

**Authors:** Krzysztof Przystupa, Yaroslav M. Chornodolskyy, Jarosław Selech, Vladyslav O. Karnaushenko, Taras M. Demkiv, Orest Kochan, Stepan V. Syrotyuk, Anatolii S. Voloshinovskii

**Affiliations:** 1 Department of Automation, Lublin University of Technology, Nadbystrzycka 38D, 20-618 Lublin, Poland; 2 Physical Faculty, Ivan Franko National University of Lviv, 79005 Lviv, Ukraineanatoliy.voloshinovskii@lnu.edu.ua (A.S.V.); 3 Faculty of Transport and Civil Engineering, Poznan University of Technology, Piotrowo 3, 60-965 Poznan, Poland; 4 Institute of Mechanical Science, Vilnius Gediminas Technical University, J. Basanavičiaus Str. 28, LT-03224 Vilnius, Lithuania; 5 School of Computer Science, Hubei University of Technology, Wuhan 430068, China; orest.v.kochan@lpnu.ua; 6 Department of Semiconductor Electronics, Lviv Polytechnic National University, Bandery Str. 12, 79013 Lviv, Ukraine; stepan.v.syrotiuk@lpnu.ua

**Keywords:** energy band structure, luminescence, exciton, light yield, scintillator

## Abstract

This study aims to determine the optimum composition of the CeBr_1−x_I_x_ compound to achieve the maximum light output. It is based on calculations of the band energy structure of crystals, specifically taking into account the characteristics of the mutual location of local and band 5d states of the Ce^3+^ ions. The band energy structures for CeBr_2_I and CeBrI_2_ crystals were calculated using the projector augmented wave method. The valence band was found to be formed by the hybridized states of 4p Br and 5p I. The 4f states of Ce^3+^ are located in the energy forbidden band gap. The conduction band is formed by the localized 5d1 states, which are created by the interaction between the 5d states of Ce^3+^ and the 4f^0^ hole of the cerium ion. The higher-lying delocalized 5d2 states of Ce^3+^ correspond to the energy levels of the 5d states of Ce^3+^ in the field of the halide Cl^0^ (Br^0^) hole. The relative location of 5d1 and 5d2 bands determines the intensity of 5d–4f luminescence. The bottom of the conduction band is formed by localized 5d1 states in the CeBr_2_I crystal. The local character of the bottom of the conduction band in the CeBr_2_I crystal favors the formation of self-trapped Frenkel excitons. Transitions between the 5d1 and 4f states are responsible for 5d–4f exciton luminescence. In the CeBrI_2_ crystal, the conduction band is formed by mixing the localized 5d1 and delocalized 5d2 states, which leads to quenching the 5d–4f luminescence and a decrease in the light output despite the decrease in the forbidden band gap. CsBr_2_I is the optimum composition of the system to achieve the maximum light output.

## 1. Introduction

Nowadays, there is a need for the mass production of reliable, highly sensitive, and cheap sensors for radiation monitoring of the environment [1], global tracking of radioactive materials [2,3], medicine [4], energy sector, Internet of Things [5,6], etc. There are known metrological methods for improving the accuracy of sensors such as calibration and diagnostics [7], technical methods such as designing new constructions [5], and mathematical methods such as data processing techniques [7]. However, these methods do not eliminate the reasons of sensor inaccuracies. Thus, deep fundamental studies are essential to develop robust sensors.

The main requirements for scintillation materials concern such scintillator parameters as light output and speed. The need for high-speed characteristics has especially increased with the need to develop scintillators to fully use the possibilities of positron emission tomography operating in the time-of-flight registration mode [8], with respect to spatial separation in medical imaging.

Recently, among many potential scintillators, attention is focused on nanocrystals of halide perovskites, which exhibit short decay times (<15 ns) and high light yields [9]. However, the challenge of compacting nanocrystals into bulk transparent samples has led researchers to turn to single crystalline objects. Certain potential for time resolution lies in lanthanide halide crystals, particularly LaBr_3_–Ce, which shows a coincidence time resolution of 77 ps [10], which is almost comparable with 51 ps of BaF_2_ [11,12], demonstrating one of the shortest luminescence decay times (0.8 ns) among crystalline scintillators. Therefore, efforts aimed at improving the parameters of the existing fast scintillators, whose growth technology was developed [13], are welcomed.

The relevance of the study presented in this work is determined by the search for fast scintillators suitable for application as detectors in positron emission tomography systems in the time-of-flight mode. This study aims to determine the optimum composition of the CeBr_3−x_I_x_ compound to achieve maximum light output. This is based on calculations of the band energy structure of crystals, in particular, considering the peculiarities of the mutual location of the local and band states of the Ce^3+^ ion.

Heavy inorganic crystals of cerium trihalide are thought to be promising scintillators for their use as radiation detectors in high-energy physics and medicine. The above-mentioned crystals gained popularity because of fast decay time constant and high light yield of 5d–4f Ce^3+^ transitions. The CeBr_3_ crystal is the most efficient among CeX_3_ (X = F, Cl, Br, I) crystals and has light yield (*LY*) > 60,000 photons/MeV and 4% energy resolution at 662 KeV [14]. The CeI_3_ crystal, which has a much smaller forbidden band gap (*E*_g_), does not have such a high luminescence efficiency, as it is expected according to the fundamental light yield limit, which is inversely proportional to the value of *E*_g_ [15]:

(1) $$LY=\frac{10^{6}}{\beta E_{g}}\;\cdot S\cdot QE$$

where *β* is a constant, *E*_g_ is a forbidden band gap of the solid, *S* characterizes the efficiency of energy transfer from the electron–hole pairs to the scintillating ions, and *QE* is the quantum efficiency of luminescence.

In addition to the search for scintillation materials with low *E*_g_, there is another approach to increase light yield related to the efficiency (*S*) of energy transfer to the luminescence center. This approach is based on the synthesis of mixed crystals [16]. The mixed crystals promote the formation of phonons with energy greater than that in original crystals, and new phonon branches for energy relaxation appear [17]. An increase in light yield was observed for (Lu,Y)AP:Ce mixed crystals with an intermediate concentration of Lu/Y [18] and for Gd_3_(Al,Ga)_5_O_12_ crystals (GAGG) with an Al/Ga ratio of 2/3 [19]. Mixed oxide scintillators LYSO demonstrate a 10–30% higher light output [20,21].

The authors of [22] also claim that, in crystals with the hybrid structure of halogens, the scintillation properties become better with the decrease in the hot electron thermalization length, which improves the *S* parameter. As the phonon energy increases, the length of electron thermalization decreases, which reduces the probability of electron capture at defects and increases the luminescence intensity. The additional phonon branches accelerate electron relaxation processes as well. In the mixed crystals, where the phonon subsystem plays an important role, it is necessary to consider the peculiarities of the structure of the electronic energy of crystals, which also affects the efficiency of luminescence processes. The reduction in the thermalization length through the cation disorder in rare-earth metal lithium halides leads to a decrease in or blocking of diffusion pathways [23]. Recent experimental studies showed that the luminescence properties of CeI_3_ and CeBr_3_ can be significantly improved by changing the concentration of substituted halogen x in the structure of CeBr_3−x_I_x_ [24]. In particular, it was shown that Sr co-doping of CeBr_3_ improves its energy resolution [25], and the light output of other halide binary compositions increases upon mixing halides [16,26]. It was also demonstrated that the halide substitution of Eu in CsCaI_3_ crystals [27] as well as in CsSrBrI_2_, CsCaBrI_2_, and CsSrClBr_2_ crystals [28] enhances the luminescent properties of such materials by modifying their structure.

Scintillators based on lanthanide halides activated with cerium were patented as early as 2008 [13]. The next step in the development of lanthanide-based scintillators is the transition to self-activated scintillators based on cerium, CeX_3_, which, similar to LaX_3_–Ce, exhibit commensurable scintillation parameters but, due to their higher atomic number Z, demonstrate higher absorption capability for ionizing radiation. Additionally, they show lower background radiation compared with LaX_3_ [29].

The idea of enhancing the scintillation parameters of cerium halide scintillators, specifically CeBr_3_, through mixed halide crystals Ce(Br,I)_3_, is inspired by the demonstrated improvement in the timing properties of the La(Br,I)_3_:Ce system [30]. This concept is also supported by numerous studies showing a general trend of improving scintillation parameters in mixed halide single crystal scintillators. For instance, in the system with the general formula of CeBr_3−x_Cl_x_, where 0 ≤ x ≤ 3, the light yield changed from 44,000 to 60,000 photons/MeV under ^137^Cs γ-ray irradiation when transitioning from CeCl_3_ to CeBr_3_. Therefore, the attempts to improve the light output in the CeBr_3−x_I_x_ system [24] are well founded. These attempts were motivated by certain features of the scintillation process that contribute to an increase in the light output. The decrease in the band gap was expected to enhance the light output by substituting bromine with iodine. Indeed, the transition from CeBr_3_ to CeBr_2_I increases the light output from 60,000 to 70,000 photons/MeV [24]. However, further increase in the iodine content resulted in a deterioration of the scintillation parameters. Based on our previous research on the energy structure of CeX_3_ crystals (X = F, Cl, Br, I) [31,32], we aim to demonstrate that the decrease in the light output is caused by peculiarities in the band energy structure of self-activated cerium halide crystals. The conduction band in these crystals is formed by the 5d states of Ce^3+^ ions. The 5d1 electrons of the Ce^3+^ ion in an electric field of a 4f^0^ hole form a conduction sub-band (5d1). It is responsible for the 5d–4f excitonic luminescence of cerium ions. The 5d electrons, in the presence of a halide hole (npX0), form a higher-lying band (5d2) with delocalized electrons. The overlap between the localized (5d1) and delocalized (5d2) states leads to significant quenching of the excitonic luminescence, which must be the cause of CeBrI_2_ luminescence quenching.

In the case of the mixed CeBr_3−x_I_x_ crystal, there is only a certain range of x values at which success can be achieved because in CeBr_3_, the bottom of the conduction band is formed by local states that promote exciton 5d–4f luminescence, whereas in CeI_3_, the bottom of the conduction band is formed by delocalized states. As a result, 5d–4f luminescence in room temperature becomes negligible [31]. Therefore, for a better understanding of the physical processes that occur after this kind of substitution, this paper presents the results of theoretical studies of the energy structure for the CeBr_2_I and CeBrI_2_ crystals, as well as the influence of the location of localized and delocalized 5d states on the luminescence efficiency.

We intend to theoretically predict the optimum composition of the CeBr_3−x_I_x_ mixture to achieve the highest light output. It is expected that reducing the forbidden band gap and appearing additional channels for electron thermalization through iodine substitution can improve the light output. We plan to demonstrate that, in addition to band-gap reduction, the energy structure of the CeBr_3−x_I_x_ conduction band will also change, leading to a decrease in the light output. The localized 5d states of cerium ions, associated with the formation of Frenkel excitons, will overlap with the delocalized states of the conduction band, resulting in luminescence quenching. We aim to show that CeBr_2_I crystals are the optimum composition for achieving the maximum output in the CeBr_3−x_I_x_ system. This is possible because CeI_3_ represents the case where delocalized and localized states strongly overlap, leading to luminescence quenching.

## 2. Modeling and Calculations

**Figure 1:**
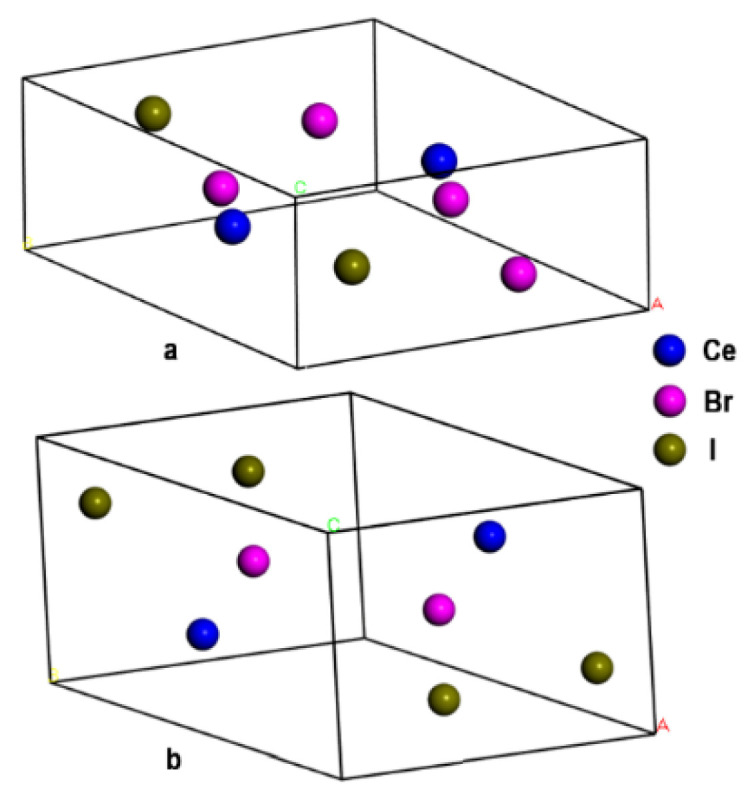
Modeled cells of CeBr_2_I (**a**) and CeBrI_2_ (**b**) crystals.

All calculations were performed within the framework of the density functional theory using Abinit v8.10 software [33] distributed under the open-source license. Lattice constants obtained using X-ray spectroscopy [24] were used as input data to calculate the main energy parameters of the CeBr_2_I and CeBrI_2_ crystals. Crystal cells (Figure 1) were modeled with Avogadro 1.91.0 software using constants and space symmetry groups shown in Table 1.

As can be seen from Table 1, the CeBr_2_I crystal has the hexagonal lattice similar to CeCl_3_, CeBr_3_, and CeF_3_. The CeBrI_2_ crystal has the orthorhombic lattice similar to CeI_3_. The symmetry of the lattice varies depending on the iodine concentration. According to the experimental data presented in [24], the structure of CeBr_3−x_I_x_ changes to orthorhombic when x > 0.5.

The projector augmented wave (PAW) method was used [34] to provide accurate representation of energy properties with respect to the fast-oscillating components of the wave functions of electrons near the cores. This method combines features of both the pseudopotential approach and the plane wave method. The connection between the wave function $|\psi_{n}\left(r\right)\rangle$ and the pseudo-wave function $|{\tilde{\psi }}_{n}\left(r\right)\rangle$ can be expressed by the formula

(2) $$|\psi_{n}\left(r\right)\rangle =|{\tilde{\psi }}_{n}\left(r\right)\rangle +\underset{a}{\sum }\underset{i}{\sum }\left(|\varphi_{i}^{a}\left(r\right)\rangle -|{\tilde{\varphi }}_{i}^{a}\left(r\right)\rangle \right)\langle {\tilde{p}}_{i}^{a}|{\tilde{\psi }}_{n}\rangle$$

where

$\varphi_{i}^{a}\left(r\right)$—atomic wave function,
$|{\tilde{\varphi }}_{i}^{a}\left(r\right)\rangle$—pseudo-wave function,
$\langle {\tilde{p}}_{i}^{a}|$—projector function.

The sum is performed out of the spheres of joining, which are numbered with the index *a*. The index *i* = {*n*, *l*, *m*} corresponds to quantum numbers—the principal, orbital, and magnetic, respectively.

From (4), it is easy to see that

(3) $$|\psi_{n}\left(r\right)\rangle =\tau |{\tilde{\psi }}_{n}\left(r\right)\rangle$$

where τ transforms the pseudo-wave function $|{\tilde{\psi }}_{n}\left(r\right)\rangle$ into the electron wave function $|\psi_{n}\left(r\right)\rangle$.

The explicit form of the τ operator is derived from (2):

(4) $$\tau =1+\underset{a}{\sum }\underset{i}{\sum }\left(|\varphi_{i}^{a}\left(r\right)\rangle -|{\tilde{\varphi }}_{i}^{a}\left(r\right)\rangle \right)\langle {\tilde{p}}_{i}^{a}|$$

The stationary Schrödinger equation is as follows:

(5) $$H|\psi_{nk}\rangle =|\psi_{nk}\rangle \epsilon_{nk}$$

which will become as follows when taking into account (3):

(6) $$\tau^{+}H\tau |{\tilde{\psi }}_{nk}\rangle =\tau^{+}\tau |{\tilde{\psi }}_{nk}\rangle \epsilon_{nk}$$

with the same desired electron spectrum $\epsilon_{nk}$ as in (7).

The electron density in the PAW method is determined by three additions:

(7) $$\rho \left(r\right)=\tilde{\rho }\left(r\right)+\underset{a}{\sum }\left(\rho^{a}\left(r\right)-{\tilde{\rho }}^{a}\left(r\right)\right)$$

The first addition is a smooth pseudo-density ${\tilde{\rho }}^{a}$, which can be described by the Fourier transform:

(8) $$\tilde{\rho }\left(r\right)=\underset{a}{\sum }f_{nk}{\left|{\tilde{\psi }}_{nk}\left(r\right)\right|}^{2}=\frac{1}{\Omega }\underset{G}{\sum }\tilde{\rho }\left(G\right)e^{iGr}$$

where

$f_{nk}$—the occupation numbers of one-electron state,
***k***—a vector of the first Brillouin zone,
*n*—the number of filled electronic bands,
Ω—the volume of the primitive lattice,
G—is a vector of the reciprocal lattice of the crystal.

For the electronic function, Formula (8) is as follows:

(9) $$\rho \left(r\right)=\underset{nk}{\sum }f_{nk}{\left|\psi_{nk}\left(r\right)\right|}^{2}=\frac{1}{\Omega }\underset{G}{\sum }\rho \left(G\right)e^{iGr}$$

There is a significant difference between Formulae (8) and (9). Equation (9) considers ~10^3^ vectors G, but achieving the same level of accuracy for Equation (8) requires ~10^6^ vectors. That is why it is impossible to solve Equation (7) using the electronic function $|\psi_{nk}\rangle$ even when embracing supercomputers.

The next two additions of electronic densities inside the augmented sphere are determined by the projected coefficients of filled states:

(10) $$W_{ij}^{a}=\underset{nk}{\sum }f_{nk}\langle {\tilde{\psi }}_{nk}|{\tilde{\rho }}_{i}^{a}\rangle \langle {\tilde{\rho }}_{i}^{a}|{\tilde{\psi }}_{nk}\rangle$$

and

(11) $$\rho^{a}\left(r\right)=\underset{ij}{\sum }W_{ij}^{a}\varphi_{i}^{a^{*}}\left(r\right)\varphi_{j}^{a}\left(r\right)$$

(12) $${\tilde{\rho }}^{a}\left(r\right)=\underset{ij}{\sum }W_{ij}^{a}{\tilde{\varphi }}_{i}^{a^{*}}\left(r\right){\tilde{\varphi }}_{j}^{a}\left(r\right)$$

The idea of the PAW method is transforming the Schrödinger equation into the form where the unknown function of state is ${\tilde{\psi }}_{nk}$. It is much less computationally expensive than the original one $\psi_{nk}$. If the function is found, using the transformation operator τ, it is easy to obtain the state function $\psi_{nk}$. The Hartree potential and electron densities can be calculated using $\psi_{nk}$.

One of the features of the lanthanide ions is the presence of highly localized states with exchange-correlation energy that cannot be correctly described by either the local density approximation (LDA) model or its generalized gradient modification (GGA) [35]. The approximation of the above-mentioned exchange-correlation interaction functionals is based on the model of the homogeneous electron gas, which, however, contradicts the localization of the electron density in narrow energy regions for the lanthanide ions. Two approaches were considered to solve this problem: the use of the hybrid functional of the exchange-correlation interaction PBE0 [36] and Hubbard corrections in the DFT + U method [37]. The former works better for crystals with a high concentration of lanthanide ions where they are constitutional ions of the matrix [32]. On the other hand, the DFT + U method is accurate enough to describe the energy states of lanthanides when computational power is limited.

All calculations were performed on the Monkhorst–Pack grid with the size of 10 × 10 × 10. The basis of wave functions was formed by plane waves with cutoff energies of 48 Ha (1306.15 eV) and 108 Ha (2938.83 eV) within the PAW sphere. For an accurate representation of the conduction band, 200 energy states of the investigated crystals were considered in the calculations.

The well-known formula was used to calculate the effective mass of electrons:

(13) $$\;m_{e}^{*}=\hbar^{2}{\left(\frac{d^{2}k}{dk^{2}}\right)}^{-1}$$

For a free particle, the dispersion law is quadratic, so the effective mass is constant and equal to the mass of rest of the electron *m*_0_. The situation is more complicated in a crystal, and the dispersion law differs from the quadratic one. Nevertheless, the dispersion law *E*(*k*) curve near the extrema is usually well approximated by a parabola, where the effective mass $m_{e}^{*}$ is also a constant, although different from *m*_0_. At the same time, $m_{e}^{*}$ can be positive (near the bottom of the conduction band) and negative (near the top of the valence band). The tensor nature of the effective mass in anisotropic crystals illustrates that in the crystal lattice, an electron moves as a quasiparticle, and its movement is controlled by the direction relative to the crystallographic axes of the crystal. However, the value of $m_{e}^{*}$ is determined not by energy but by the state, which is based on vector *k*. In this work, the effective mass was obtained after post-processing of the calculated energy band structure. Abipy software 0.8.0 was used to analyze the raw output data of Abinit v8.10, which allows calculating various physical characteristics based on the information about the energy structure of the crystal using Python programming language. The developed software allowed us to estimate the value of the effective mass of charge carriers at any pre-calculated energy level in the proximity of a given *k*-point performing approximation by a paraboloid in a three-dimensional *k*-space. All presented values of $m_{e}^{*}$ in this paper were obtained around the highly symmetrical point Γ, in which, for these cases, the energy of the conduction band reaches its minimum.

## 3. Results and Discussions

**Figure 2:**
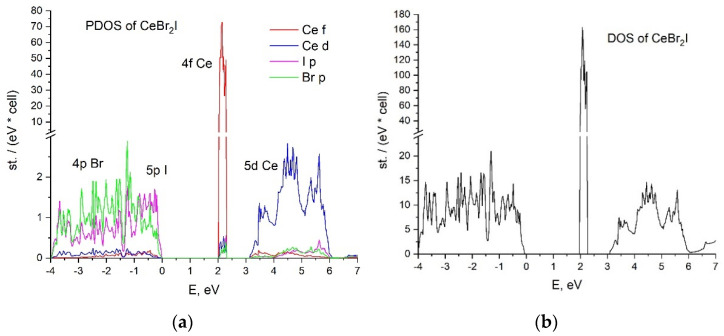
CeBr_2_I crystal (**a**) partial density of states; (**b**) total density of states.

**Figure 3:**
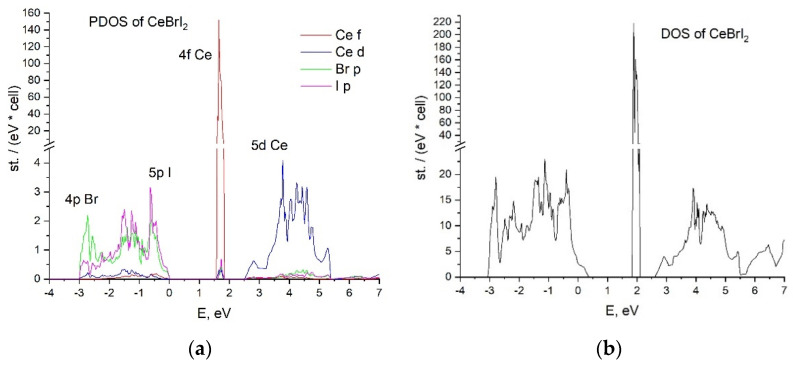
CeBrI_2_ crystal (**a**) partial density of states; (**b**) total density of states.

The calculated partial and total densities of states of the CeBr_2_I and CeBrI_2_ crystals are presented in Figure 2 and Figure 3, respectively. The top of the valence band in both cases is formed by the hybridized states of 4p Br and 5p I. The valence bandwidth of the CeBr_2_I crystal is 4 eV, which is 1 eV larger than in the case of CeBrI_2_.

There are 4f cerium states in the middle of the forbidden zone. Their peaks of 0.3 eV width are located above the valence band by 2 eV for CeBr_2_I and 1.6 eV for CeBrI_2_.

The conduction band is formed by 5d Ce states in both cases, demonstrating different distributions of the density of states. The contribution of 4p Br- and 5p I- states to the conduction band is negligible.

**Figure 4:**
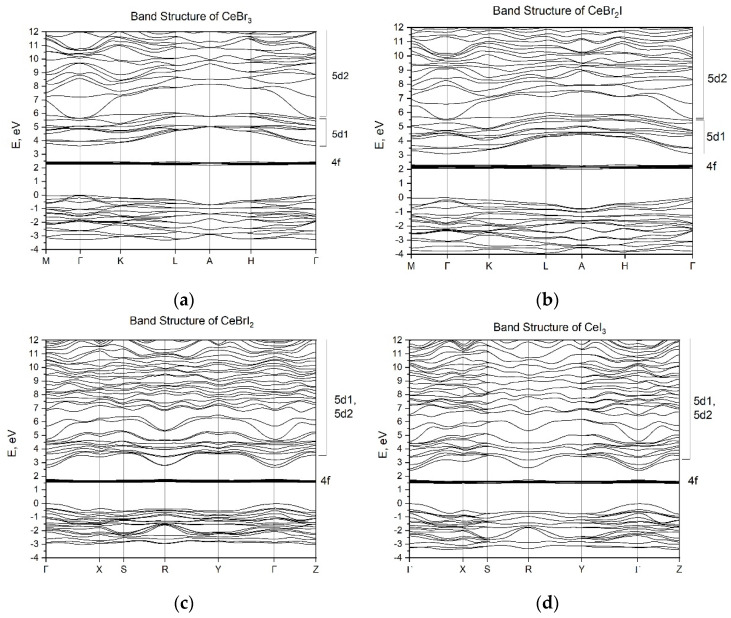
The energy band structure of CeBr_3−x_I_x_ crystal: (**a**) CeBr_3,_ (**b**) CeBr_2_I, (**c**) CeBrI_2_, (**d**) CeI_3_.

The energy band structures of the CeBr_2_I and CeBrI_2_ crystals are presented in Figure 4. The energy parameters of the bands were calculated along the high-symmetry paths of the Brillouin zone: Γ-K-L-A-H-Γ for CeBr_2_I and Γ-X-S-Y-Γ-Z for CeBrI_2_. The energy band structures of the CeBr_3_ and CeI_3_ crystals [31] are presented for comparison.

The clear localization of 4f-states and the formation of localized (5d1) and delocalized (5d2) 5d-states of cerium ions in the conduction band, as shown for CeBr_3_ (see Figure 4a) [31], are features of the electronic structure of the self-activated CeX_3_ scintillators. Conclusions about the peculiarities of 5d-state localization in the conduction band were drawn based on the difference between the effective masses of electrons in the bottom of the conduction band and electrons in the depth of the conduction band. Additional evidence for the existence of the local 5d1 states of cerium ions in self-activated CeX_3_ is the presence of typical luminescence of the cerium 4f–5d ions. In the CeBr_2_I crystal, the energy sub-bands 5d1 and 5d2 with different effective masses of electrons ($m_{e}^{*}$ (5d1) = 1.99 *m*_0_ and $m_{e}^{*}$ (5d2) = 0.58 *m*_0_) can also be distinguished. The width of the 5d1 sub-band at Γ-point is the splitting of the 5d states of cerium ions by the crystal field of the matrix. Excitation of electrons in the 5d2 sub-band does not necessarily lead to luminescence, as the high mobility of electrons leads to their significant diffusion, which negatively affects the efficiency of their recombination with holes [31,32]. The energy gap between the top of the halide valence band and the 5d2 sub-band of the conduction band is considered as the value of the forbidden band gap (*E*_g_). The parameters of the energy bands of the CeBr_3_, CeBr_2_I, CeBrI_2_, and CeI_3_ crystals are given in Table 2. The table also shows the position of 4f-cerium levels relative to the top of the valence band (*E*_4f_) and the energy of 4f–5d transitions (*E*_4f–5d_). The energy of the forbidden band gap for CeBr_3_ (*E*_g_ = 5.7 eV) agrees well with the experimental data for the LaBr_3_ crystal (*E_g_* = 5.9 eV), where the conduction band is formed by delocalized 5d-states. However, for the CeI_3_ crystal, the band gap (*E*_g_ = 2.4 eV) is underestimated in comparison with the experimental results (*E*_g_ = 3.8 eV) [31]. The results for the energy of 4f–5d transitions for all crystals are also underestimated. Since the experimental data for *E*_g_ in CeBr_2_I and CeBrI_2_ crystals are not available, the calculated results of *E*_g_ = 5.4 eV and *E*_g_ = 2.6 eV should be considered as approximate values. Such underestimation of the energy parameters is a well-known problem for DFT calculations [38]. Other theoretical approaches (such as the Green function method) can provide a more accurate value of the band gap, but the positions of 4f states will be less precise. However, the used method allowed us to determine the features of the structure of the conduction band such as the existence of localized and delocalized 5d states of cerium. The presence of localized states at the bottom of the conduction band allows us to apply the model of Frenkel self-trapped excitons to explain the 5d–4f luminescence of cerium ions. The increase in the overlap of localized and delocalized states with increasing iodine concentration explains the tendency of light yield decrease (CeBrI_2_, CeI_3_) due to the decrease in quantum efficiency (*QE*). The calculation method also shows a general trend of decrease in the band gap with the increase in iodine concentration, which explains the increase in the CeBr_2_I light yield compared with CeBr_3_.

With respect to the peculiarities of the conduction band of CeX_3_ crystals, the conduction band of the CeBrI_2_ crystal (Figure 4c) shows similar characteristics to CeI_3_ (Figure 4d). The dispersion of the bottom of the conduction band with respect to the wave vector k is higher in the case of CeBrI_2_ compared with CeBr_2_I and CeBr_3_. It is characterized by a small effective electron mass of 1.03 *m*_0_, which is typical for delocalized conduction band states. By analyzing the dependence of the forbidden energy band gap on the concentration of iodine ions, it can be noted that it decreases with the increase in iodine concentration in the following direction:

(14) $${\mathrm{CeBr}}_{3}\to {\;\mathrm{CeBr}}_{2}I\to {\;\mathrm{CeBrI}}_{2}\to {\;\mathrm{CeI}}_{3}$$

This series in a descending order of the band gap allows us to see the general trend of the influence of the substitution of the bromide ions with the iodine ones on the forbidden band gap of the crystal. Comparing the luminescence properties of the CeBr_3_, CeBr_2_I, CeBrI_2_, and CeI_3_ crystals according to the fundamental light yield limit (1), which is inversely proportional to the band gap, it is expected that the scintillation efficiency will increase in the order shown in (14). Indeed, as mentioned above, the transition from CeBr_3_ to CeBr_2_I leads to the increase in light yield from 40,000 to 70,000 photons/MeV, consistent with the dependence of light yield on *E*_g_. In the case of the mixed CeBr_2_I crystal, light output increases not only due to a decrease in *E*_g_ but also due to an increase in the efficiency of energy transfer to the luminescent centers (*S*) because of the presence of additional phonon branches. This contributes to faster relaxation of electrons in the conduction band.

**Figure 5:**
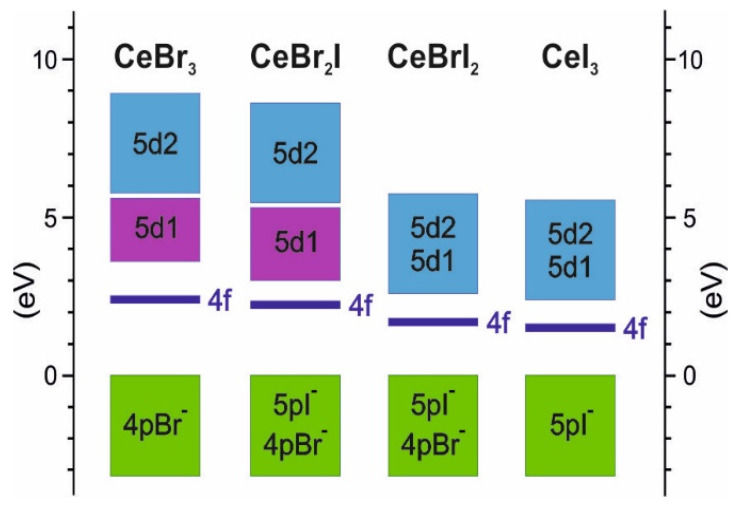
Schemes of calculated energy band positions for CeBr_3−x_I_x_.

Further increase in the concentration of iodine leads to the decrease in the band gap width, which should increase the intensity of the luminescence light. However, in practice, the situation is opposite; for CeBrI_2_, *LY* is 50,000 photons/MeV, and for CeI_3_, it is 5000 photons/MeV. To explain this dependence, it is necessary to analyze the position of the local 5d1 state relatively to the delocalized 5d2 one. The schemes in Figure 5 show that in contrast to CeBr_3_ (Figure 4a) and CeBr_2_I (Figure 4b) crystals, the 5d1 and 5d2 states in CeBrI_2_ and CeI_3_ crystals overlap, and the bottom of the conduction band is formed by delocalized states with the effective mass of electrons of 1.03 *m*_0_ for CeBrI_2_ and 0.26 *m*_0_ for CeI_3_. Taking into account these small effective masses, the bottom of the conduction band will be formed by delocalized states. The 5d→4f luminescence from such states should be very weak or completely absent, demonstrating significant temperature dependence of the luminescence intensity. Therefore, for CeBrI_2_ and CeI_3_ crystals, the values of the light yield are determined by the low quantum efficiency (*QE*) of the luminescence process.

The outcome of our research reveals specific characteristics of the 5d conduction band in CeX_3_ crystals. The lower sub-band, labeled as 5d1, is formed by the energy states of a 5d electron in the presence of a 4f0 hole. The upper sub-band, labeled as 5d2, is formed by the energy states of a 5d electron in the electric field of the npX0 halide. The 5d1 sub-band is characterized by a higher effective mass of electrons compared with the effective mass in the 5d2 band. The larger effective mass promotes electron localization within a time scale of 550 fs [39], leading to the formation of self-localized Frenkel excitons. This allows us to consider the energy states of the 5d1 sub-band as localized. This is supported by the spectral structure of the excitation luminescence of cerium halides. The excitation spectrum range attributed to 4f–5d1 transitions is structured and corresponds to the number of components into which the 5d states are split by the crystal field. Transitions from 4f states to delocalized states in the 5d2 conduction sub-band do not exhibit any structure, and the excitation spectrum appears as a smooth (unstructured) curve. Such spectral characteristics (structured for 4f–5d1 and unstructured for 4f–5d2) are typical for the 5d–4f excitation spectrum in the luminescence of CeF_3_ crystals within this family [32,40]. The structuring of the conduction band into localized states (5d1) and delocalized states (5d2) determines the efficiency of the luminescent process in the series of CeX_3_ compounds. The 5d–4f luminescence is efficient when the bottom of the conduction band in CeBr_3_ is formed by localized states, as is the case of CeBr_3_. Conversely, this luminescence is less efficient when these sub-bands significantly overlap, as is the case of CeI_3_.

**Table 2:** Parameters of the energy structure of the CeBr_3−x_I_x_ crystals. *E*_g_—The energy band gap; *E*_4f_—the position of 4f states above the top of the valence band; *E*_4f–5d_—the energy of 4f–5d transition.

		CeBr_3_	CeBr_2_I	CeBrI_2_	CeI_3_
*E*_g_, eV	Calculation	5.7	5.4	2.6	2.4
	Experiment	5.9 [41]	–	–	3.8 [41]
*E*_4f_, eV	Calculation	2.3	2.2	1.7	1.6
	Experiment	1.1 [41]	–	–	1.0 [41]
*E*_4f–5d_, eV	Calculation	1.3	1.2	0.9	0.7
	Experiment	3.5 [24]	3.1 [16]	2.8 [16]	2.7 [31]

## 4. Conclusions

The PAW method and the hybrid exchange-correlation functional allow us to calculate the energy structure of halide crystals with lanthanide ions and to explain the energy structure formation and peculiarities of the CeBr_3−x_I_x_ luminescence energy parameters of these crystals.

The valence bands of CeBrI_2_ and CeBr_2_I crystals are formed by the hybridized 4p Br and 5p I states. The 4f cerium states are localized within the band gap. The bottom of the CeBr_2_I conduction band, as in CeBr_3_, is created by the local 5d states forming 5d1 sub-band with an effective mass of charge carriers of 1.99 *m*_0_. The large effective mass of 5d1 sub-band carriers promotes the localization of electronic excitation with the appearance of the Frenkel self-trapped exciton. Its luminescence corresponds to the 5d–4f transition in the Ce ion. The delocalized 5d2 states with small effective mass are placed above the local 5d1 states in CeBr_3_ and CeBr_2_I crystals and significantly overlap in CeBrI_2_ and CeI_3_ crystals.

As the iodine concentration in CeBr_3_ and CeBr_2_I crystal series increases, the band gap decreases. This enhances the light yield in CeBr_2_I compared with that in CeBr_3_. However, further increase in iodine concentration in CeBrI_2_ and CeI_3_ decreases the quantum efficiency of luminescence and neutralizes the positive effect from band-gap reduction and the decrease in the electron thermalization length that results in the light yield decrease. Our research clearly demonstrates the optimum composition of the system for achieving the maximum light output, thereby allowing researchers to focus on improving the compound of CeBr_2_I.

## Figures and Tables

**Table 1:** Crystal lattice parameters and space groups for the CeBr_2_I and CeBrI_2_ crystals.

Crystal	a, Å	b, Å	c, Å	Space Group
CeBr_2_I	8.046	8.046	4.465	P63/m
CeBrI_2_	4.244	18.18	9.444	Cmmm

## Data Availability

The data presented in this study are available on request from the corresponding author.
